# Spontaneous closure of an extensive postdebridement perineal wound in a newly diagnosed diabetic patient presenting with necrotizing fasciitis

**DOI:** 10.1002/ccr3.2805

**Published:** 2020-03-20

**Authors:** David Muchuweti, Edwin Muguti, Simbarashe Gift Mungazi

**Affiliations:** ^1^ Department of Surgery College of Health Sciences University of Zimbabwe Harare Zimbabwe; ^2^ Department of Surgery College of Sciences University of Zimbabwe Harare Zimbabwe; ^3^ Department of Surgery National University of Science and Technology Bulawayo Zimbabwe

**Keywords:** debridement, diabetes mellitus, Fournier' s gangrene, glycemic control, immunosuppression, necrotizing fasciitis, spontaneous

## Abstract

Diabetes mellitus may present for the first time with necrotizing fasciitis. Early treatment of septic shock and immediate surgical debridement reduces mortality. A diverting loop colostomy prevents soiling of extensive postdebridement wound. Local wound care together with good glycemic and infection control leads to spontaneous wound closure.

## Graphical abstract

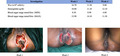

## 1 INTRODUCTION

Necrotizing fasciitis is a rapidly progressive deep soft tissue infection, which starts in the superficial fascia and moves rapidly along fascial planes.^1^ The necrotizing fasciitis is common in the perineum and is referred to as Fournier's gangrene but may occur in any part of the body.^2^ When left unabated, the disease is progressive and accompanied by severe systemic toxicity^3^ and high mortality.^4^ Risk factors for mortality include location of necrotizing fasciitis in the perineum or trunk, diabetes mellitus, and delay in diagnosis.^5^

Diseases that cause immunosuppression such as diabetes mellitus, renal failure, malnutrition, HIV infection, and peripheral vascular disease are risk factors.^6^ Necrotizing fasciitis occurs in 11% of diabetic patients.^7^, ^8^ The trigger of Fournier's gangrene includes trauma, skin infections, instrumentation, surgery, scratch, and trauma from catheterization,^9^ and in females, infections such as Bartholin's abscess. Necrotizing fasciitis is commonly polymicrobial by both aerobes and anaerobes, and patients need adequate antibiotic cover.^10^, ^11^ The classification of necrotizing fasciitis into types 1‐3 is based on the microbiology ^10^, ^12^

Survival depends on early diagnosis and treatment. A high index of suspicion is required, and diabetes mellitus must be excluded in patients presenting with Fournier's gangrene. Cardiovascular collapse and multisystem failure may occur^3^ if diagnosis and treatment is delayed. The mainstay of successful treatment lies in fluid resuscitation, antibiotic treatment, and surgical debridement^13^ and control and treatment of the underlying disease. Control of wound infection and glycemic control often lead to spontaneous closure of extensive postdebridement wounds, as happened in our case, thereby sparing patients from more complex types of wound closures.

## 2 CASE REPORT

A 46‐year‐old female patient was brought to our Accident and Emergency Department with a 5‐day history of a painful perianal and perineal swelling, which was increasing in size each day and discharging pus. This was preceded by perineal scratching for itchiness. A day prior to presentation, the pain had increased in intensity and the patient had difficulty in passing stool.

She had asthma and hypertension, which were well controlled. She was on salbutamol 4 mg three times a day and used becotide inhaler for her asthma and nifedipine 20 mg twice a day for her blood pressure. Her recent human immunodeficiency virus (HIV) test was negative. There was no family history of diabetes or malignancies. She was married with one child, and she neither drank alcohol nor smoked cigarettes.

**FIGURE 1:**
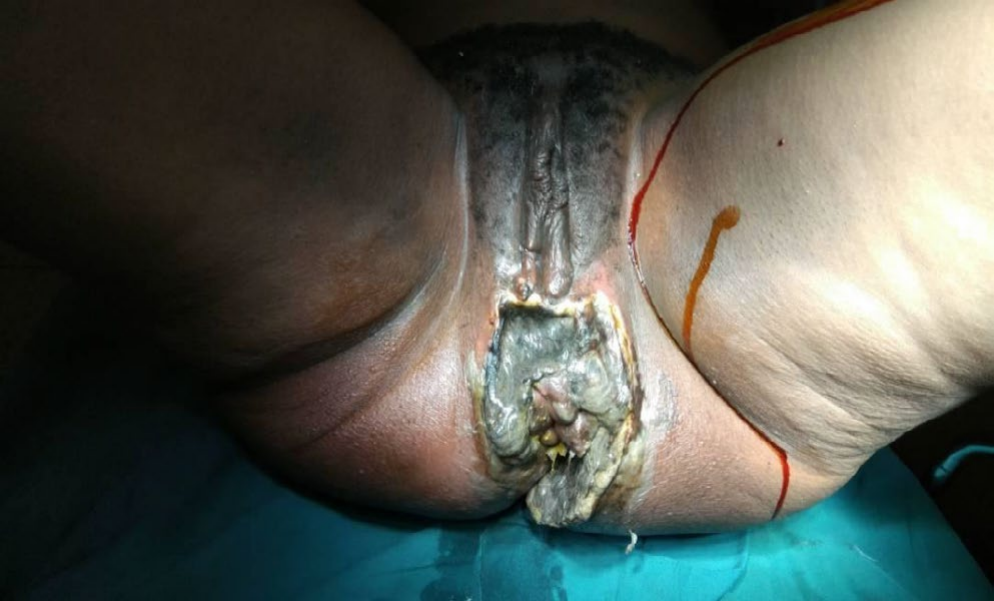
Pre‐op. extensive perineal and perianal tissue necrosis

On examination, she was ill‐looking and in pain. She was hypotensive with a blood pressure of 85/35 mmHg, a pulse rate of 110 beats per minute, and respiratory rate of 30 breaths per minute. She had an elevated temperature of 38 degrees Celsius and a low oxygen saturation of 88%. Examination of her perineum and perianal area showed a huge mass with extensive necrosis and oozing pus from its surface. This is shown in her preoperative image below (Figure 1).

Crepitus was elicited in the tissues adjacent to the ulcer. There was exquisite tenderness on digital rectal examination. Examination of other systems was normal. A random blood sugar test done showed a high blood sugar of 18.05 mmol/L. A diagnosis of necrotizing fasciitis was made in a patient with newly diagnosed diabetes mellitus.

**FIGURE 2:**
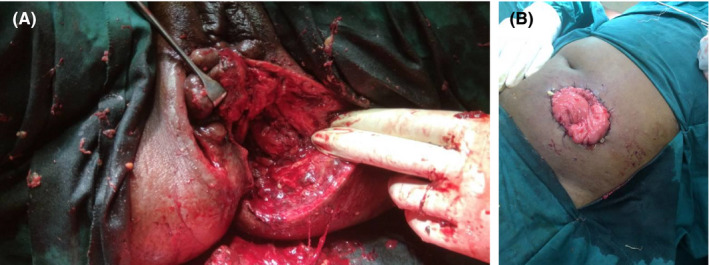
A, B, Extensive postoperative wound (2A) and diverting loop colostomy (2B)

The patient was very sick and in septic shock from fulminant sepsis. She needed high dependency unit (HDU) admission for septic shock treatment before surgery. Aggressive fluid resuscitation, antibiotic treatment, and insulin per sliding scale for glycemic control were commenced. Empirical antibiotics used were ceftriaxone 1g daily and metronidazole 500 mg intravenous 8 hourly. Her preoperative investigations are shown in Table 1 below. She had a markedly elevated white cell count. After 48 hours of resuscitation and monitoring, the patient's blood pressure improved to an average of 120/80 mmHg. Pulse and temperature came down to 100 beats per minute and 37.6 degrees Celsius, respectively. The patient was taken to theater for debridement and diverting loop colostomy as shown in Figure 2A and B, respectively. Specimens were taken for microscopy, culture, and sensitivity at the time of surgical debridement. Her preoperative investigations are shown in table below (Table 1).

**Table 1:** Preoperative investigations

Parameter	Result	Range
White cell count	31.4 × 10^9^	4 −11 × 10^9^ cells/L
Hemoglobin	11.8 g/dL	12‐16 g/dL
Platelets	417 × 10^9^	150‐450 × 10^9^
Sodium	135 mmol/L	130‐135 mmol/L
Urea	6.8 mmol/L	2.5‐5.5 mmol/L
Repeat HIV test	Negative	

**FIGURE 3:**
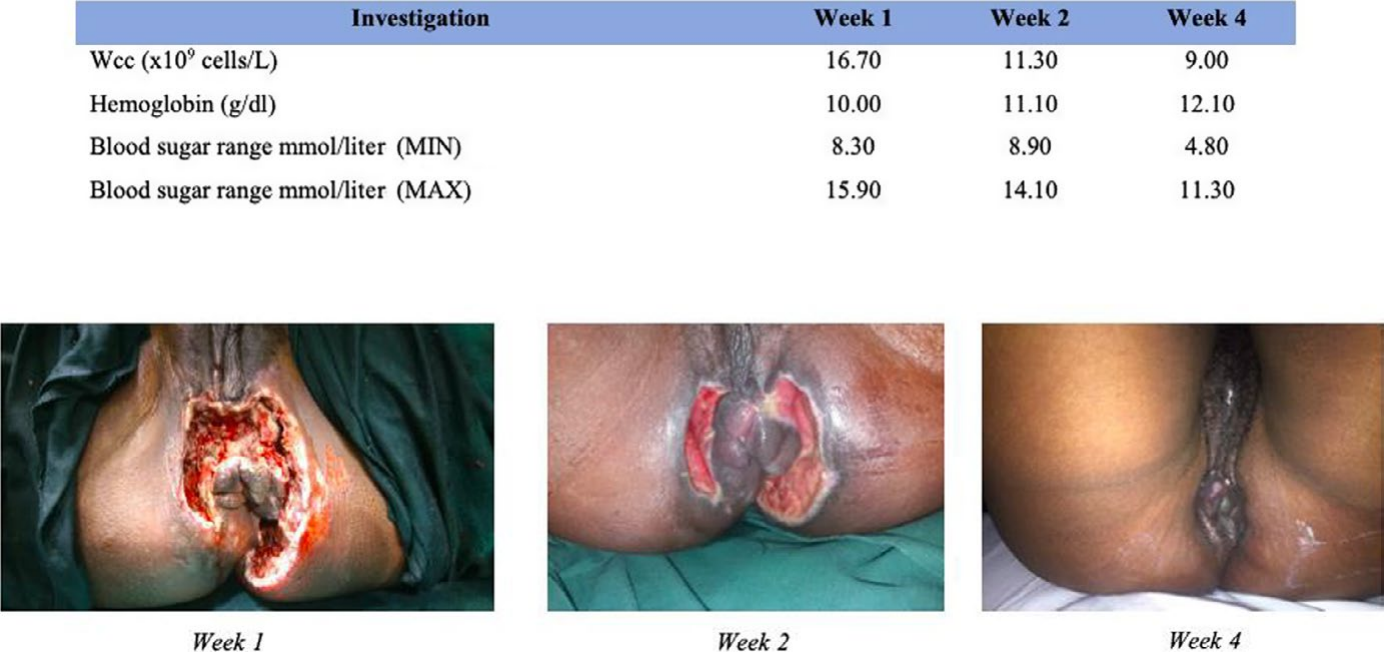
Reduction of wound size and improvement of Wcc, Hb, and blood sugar levels

Postsurgery, the patient was admitted in HDU for continued fluid resuscitation, antibiotic, analgesic, and oxygen therapy. Due to good progress, she was discharged to the surgical ward on day 3. The microbiology results grew coagulase‐negative Staphylococcus aureus sensitive to vancomycin and Klebsiella pneumoniae sensitive to amikacin. She was commenced on vancomycin 500 mg and amikacin 300 mg intravenous 8 hourly. Treatment with negative pressure wound therapy (NPWT) or vacuum‐assisted cure (VAC) was too expensive for the patient. As an alternate to wound care, she had salt sitz baths twice a day followed by dressings with silver sulfadiazine. We noted a positive correlation between rising hemoglobin (Hb) levels, decreasing white cell count (Wcc), and glycemic control and wound size as shown in Figure 3. By week 4, the wound had spontaneously closed and the patient did not need operative wound closure.

At 8 weeks, she had an uneventful reversal of colostomy. She takes glibenclamide 5 mg twice a day for her diabetes in addition to diabetic diet. She has since been seen twice in the surgical outpatient department and did not have any complaints. She has been put under the care of physicians for her diabetes, asthma, and hypertension. The figure below (Figure 3) shows reduction of wound size and improvement of Wcc, Hb, and blood sugar levels.

## 3 DISCUSSION

Fournier's gangrene was first described by Fournier in 1883.^2^ Though it is rare in females and more common in males,^12^ female gender is a risk factor for mortality.^14^

Diseases that cause immunosuppression such as diabetes mellitus^6^ and HIV infection^8^ are risk factors for Fournier's gangrene. The hyperglycemia occurring in diabetes mellitus creates a good medium for bacterial growth by providing an environment of low oxygen tension and rich substrate.^15^ Minor trauma^9^ occurred in our patient due to scratching triggered sepsis. The sepsis that followed worsened the hyperglycemia creating a vicious cycle that leads to the rapid spread of necrotizing fasciitis via tissue planes.

Diagnosis of Fournier's gangrene is clinical. Radiological examinations confirm the extent of the necrosis.^16^, ^17^ The diagnosis of Fournier's gangrene must be considered in the presence of intense pain, fever, swelling, and tenderness.^12^ Our patient had these clinical features, and the finding of crepitus on examination confirmed the diagnosis of Fournier's gangrene. Though imaging studies are important in defining the extent of necrosis prior to surgery,^16^, ^17^ our patient was too sick to have imaging studies. Imaging studies must not delay patient management as delays outweigh the benefits. Laboratory investigations are important for complete patient workup and prognostication. Fournier's gangrene favors anaerobic bacterial growth. These organisms are fastidious and are difficult to grow if proper collection and laboratory culture methods are not used.^23^, ^24^ The correct choice of antibiotics is an important adjunct to sepsis control and depends on microbiology results Necrotizing fasciitis is common in patients with diabetes mellitus ^7^, ^8^ and HIV infection.^8^ Ngugi P et al in a study on Fournier's gangrene done in December 2014 in Kenya found that the prevalence of Fournier's gangrene in HIV‐infected patients and diabetic patients was 16.4% and 11%, respectively. Our patient was HIV‐negative, and Fournier's gangrene led to the new diagnosis of diabetes mellitus. Identification and management of risk factors is important. Our patient was taking becotide, an inhaled corticosteroid (ICS) with strong anti‐inflammatory effects that are beneficial in asthma. ICS is associated with increased risk of developing diabetes mellitus and worsening glycemic control in patients with known diabetes mellitus.^20^, ^21^ However, the use of ICS in normal doses does not affect cell‐mediated immunity.^22^

A number of scoring systems have been developed to aid in the diagnosis, assessment of severity, and prognosis. The Fournier's gangrene severity index (FGSI) assesses the severity of the disease and uses nine parameters, namely temperature, heart rate, respiratory rate, serum sodium and potassium, creatinine and bicarbonate levels, hematocrit, and leukocyte count. Patients with an FGSI score (FGSIS)> 9 have a 75% probability of death, and those with FGSIS ≤9 have a 78% probability of survival.^18^ Our patient had a FGSI score of 10, which is the reason why she was admitted in HDU for preoperative management. Early adequate treatment of septic shock, sepsis source control by surgery, correct choice of antibiotics to treat infection, and good glycemic control are associated with improved survival.^1^, ^3^, ^11^ In stable patients, the burden of sepsis is reduced by surgical debridement done under local anesthesia simultaneously with fluid resuscitation soon after admission. Patients in septic shock need HDU admission for treatment before surgical debridement. Following surgical debridement, patients with extensive postdebridement perineal wounds need a diverting loop colostomy at the same sitting. A diverting colostomy reduces wound soiling in patients with open perineal wounds.^14^ A rapid clinical improvement follows when all necrotic and poorly perfused tissues are removed.^1^ The correct choice of antibiotics was made after Klebsiella pneumoniae and coagulase‐negative Staphylococcus aureus sensitive to amikacin and vancomycin, respectively, were cultured from specimens taken at the time of surgery. Our patient could not afford the cost of NPWT. We resorted to the use of salt sitz baths twice a day followed by dressing with silver sulfadiazine cream. Though accelerated wound healing has been observed with NPWT,^19^ the alternative wound care option we chose was equally good. Infection and glycemic control were predictors of wound closure. A positive correlation, as illustrated in Figure 3, was noted between wound size and rising hemoglobin levels, and glycemic and infection control.

## 4 CONCLUSION

Necrotizing fasciitis is a life‐threatening condition because of the attendant overwhelming sepsis arising from the infected site. Diabetes may present for the first time with necrotizing fasciitis. Clinicians must swiftly intervene to reduce burden of sepsis from the source even if this entails simultaneous resuscitation with sequential debridement even in the absence of NPWT. Resuscitation with good glycemic and infection control together with multiple sessions of local wound care generally results in spontaneous closure of extensive postdebridement wounds, thereby saving patients from more complex types of wound closures.

## CONFLICT OF INTEREST

None declared.

## AUTHORS' CONTRIBUTION

DM: involved in the case report design, subject research, consent, editing, and writing. EM: involved in the case report design, subject research, and writing. SGM: involved in the case report design, subject research, editing, and writing.
